# Effects of air pollution on emergency visits for acute otitis media among children: a case-crossover study in Chongqing, China

**DOI:** 10.3389/fpubh.2023.1195660

**Published:** 2023-10-16

**Authors:** Ling Xiao, Shuping Su, Cheng Chen, Hongbing Yao, Ling Ding

**Affiliations:** ^1^Department of Otolaryngology-Head and Neck Surgery, Children’s Hospital of Chongqing Medical University, Chongqing, China; ^2^National Clinical Research Center for Child Health and Disorders, Chongqing, China; ^3^Ministry of Education Key Laboratory of Child Development and Disorders, Chongqing, China; ^4^Chongqing Key Laboratory of Pediatrics, Chongqing, China; ^5^Chongqing Higher Institution Engineering Research Center of Children’s Medical Big Data Intelligent Application, Chongqing, China

**Keywords:** air pollution, air quality index, particulate matter, acute otitis media, children, case-crossover

## Abstract

**Background:**

Many epidemiological studies have demonstrated the short-term effects of air pollution on acute otitis media (AOM) in children, but few studies have explored the association between AOM and air pollution in Chinese children. This study aimed to analyze the effects of air pollution on emergency visits for AOM among children through a time-stratified case-crossover design in Chongqing, China.

**Methods:**

The outpatient medical records of children from nine main urban districts who presented with AOM between December 22, 2018 and December 21, 2021 were collected from the Children’s Hospital of Chongqing Medical University. Data for air pollution variables, including the air quality index (AQI), particulate matter ≤ 10 μm (PM_10_), PM_2.5_, SO_2_, CO, NO_2_ and O_3_ from 17 monitoring sites were collected. Data for meteorological factors as confounding variables also were collected. Conditional logistic regression was used to analyze the data with single-pollutant models, multi-pollutant models, and stratified analyses.

**Results:**

Increases in AQI, PM_10_, PM_2.5_, SO_2_, CO and NO_2_ were positively associated with emergency visits for AOM among children in single-pollutant models and stratified analyses. Increases in PM_10_, SO_2_, CO and NO_2_ were positively associated with emergency visits for AOM among children in multi-pollutant models. NO_2_ had the most statistically significant OR values in all models, whereas significant effects of O_3_ were observed only in seasonal stratification. In single-pollutant models, we found that the best lag periods were lag 0–7 for air pollution variables except for O_3_ and the largest OR values were 1.185 (95%CI: 1.129–1.245) for SO_2_ in single-pollutant models. In stratified analyses, there were no difference between groups in these statistically significant OR values through gender and age stratification, while the differences between seasons in these OR values of PM_10_, SO_2_, CO, NO_2_ and O_3_ were statistically significant. Children aged 0 years and 3–5 years represented the most susceptible population, and among the seasons, susceptibility was greater during Winter and Spring.

**Conclusion:**

Short-term exposure to air pollution can increase emergency visits for AOM among children in Chongqing, China.

## 1. Introduction

Acute otitis media (AOM) is one of the most common emergencies encountered in pediatrics and otolaryngology departments. It is an acute infectious inflammation of the mucous membrane of the middle ear, with sudden earache as the main manifestation, and in some children, AOM may be accompanied by tinnitus, hearing loss, ear discomfort, and ear discharge. If timely treatment is not administered, AOM can cause tympanic membrane perforation, hearing loss, or even chronic suppurative otitis media, which can create heavy burden for children and their families (1). The anatomical structure of children’s middle ears is smaller and shorter than that of adults, and the Eustachian tube is more neatly arranged horizontally. Accordingly, the incidence of AOM is higher in children than in adults (2). Globally, more than 80% of children under the age of 3 years have suffered from otitis media, and 30%–45% of them have experienced two or more episodes of AOM (3).

Otitis media is a multifactorial disease, with known risk factors including infection, Eustachian tube dysfunction, allergies, immunological disorders, gastroesophageal reflux, and various environmental factors. However, due to their immature immune system and lungs, children have a higher respiratory rate and spend more time breathing through their mouths than adults (4). These factors combined with their participation in more outdoor activities make them more sensitive to air pollution exposure. Indeed, the effects of air pollution on children’s otitis media have received increasing attention from researchers. One pathogenic mechanism involves interaction of pollutants with the Eustachian tube epithelium upon entry into the nasopharynx, which can directly cause swelling of the Eustachian tube mucosa, resulting in Eustachian tube stenosis (5). Second, pollutants may interfere with the clearance of mucocilia, which also can lead to Eustachian tube dysfunction (6). Eustachian tube dysfunction and stenosis ultimately lead to middle ear infection and effusion (7, 8).

Many epidemiological studies around the world have demonstrated the short-term and long-term effects of air pollution on AOM in children (9–13). However, few studies have explored the association between AOM and air pollution in Chinese children. Two birth cohort studies conducted in Changsha, China confirmed the effects of NO_2_, particulate matter ≤10 μm (PM_10_) and SO_2_ on the onset of early AOM in children (14, 15). Additionally, a limited number of Chinese reports describe analyses of the short-term correlation between air pollution and AOM in China through simple cross-sectional studies (16–18). However, these studies did not control for confounding factors such as meteorological factors and personal characteristics, nor did they consider lag effects. Therefore, the present study aimed to analyze the effects of air pollution on emergency visits for AOM among children after controlling for meteorological factors and personal characteristics through a case-crossover design.

## 2. Materials and methods

### 2.1. Study region

Chongqing is located in southwest China and is one of the four municipalities directly under the Central Government of China. As an important industrial city in the upper reaches of the Yangtze River, it is among the most heavily polluted cities in the country. Starting in 2013, Chongqing become one of the first 74 cities to implement the new air quality standard in China. The monitoring sites in Chongqing could real-time monitor the concentrations of PM_10_, PM_2.5_, SO_2_, NO_2_, O_3_, and CO.

At present, Chongqing has nine main urban districts, including the Yuzhong District, Dadukou District, Jiangbei District, Shapingba District, Jiulongpo District, Nanan District, Beibei District, Yubei District, and Banan District. The Children’s Hospital of Chongqing Medical University is the only public children’s hospital in Chongqing and is currently the third ranked children’s hospital in China. It has two hospital locations in Yubei District and Yuzhong District in Chongqing, and the average number of outpatient visits in the otolaryngology department daily ranges from 900 to 1,100. Because this hospital is the most popular choice for the treatment of children with AOM in this urban area, a large sample of cases treated at this hospital can be considered representative.

### 2.2. AOM visits

The outpatient medical records of children treated for AOM between December 22, 2018 and December 21, 2021 were collected from the Children’s Hospital of Chongqing Medical University. Children from all nine main urban districts of Chongqing were included according to the following criteria: (1) age 0–18 years; (2) main complaint of ear pain, ear discharge, ear tightness, ear discomfort, hearing loss or other related symptoms; (3) residential address in one of the nine main urban districts; (4) diagnosis of AOM according to the 10th edition (ICD-10) codes H65.0 (acute serous otitis media), H65.1 (other acute nonsuppurative otitis media), or H66.0 (acute suppurative otitis media); and (5) first visit for AOM was in our hospital. Because the course of AOM is 1–2 weeks, review within 2 weeks or multi-department visits for the same disease was only counted as one visit, and the first visit date was taken as the basis.

The following exclusion criteria were applied: (1) prior treatment of AOM in another hospital, or only review in our hospital; (2) absence of symptoms related to AOM, with otitis media found incidentally during physical examination or for another reason; or (3) AOM referred to as previous disease during hospital visit. These types of cases were excluded due to the inaccuracy of the onset date, which could affect the results of the study.

The following data were collected for each included case: patient serial number, sex, date of birth, date of visit, residential address, and chief complaint. The total number of daily cases was calculated.

The research protocol was approved by the Medical Ethics Committee of our hospital, and the research team was committed to protecting patient privacy. Because this study retrospectively collected data from medical records, consent from the patients’ guardians was not required.

### 2.3. Pollution and meteorological data

**Figure 1:**
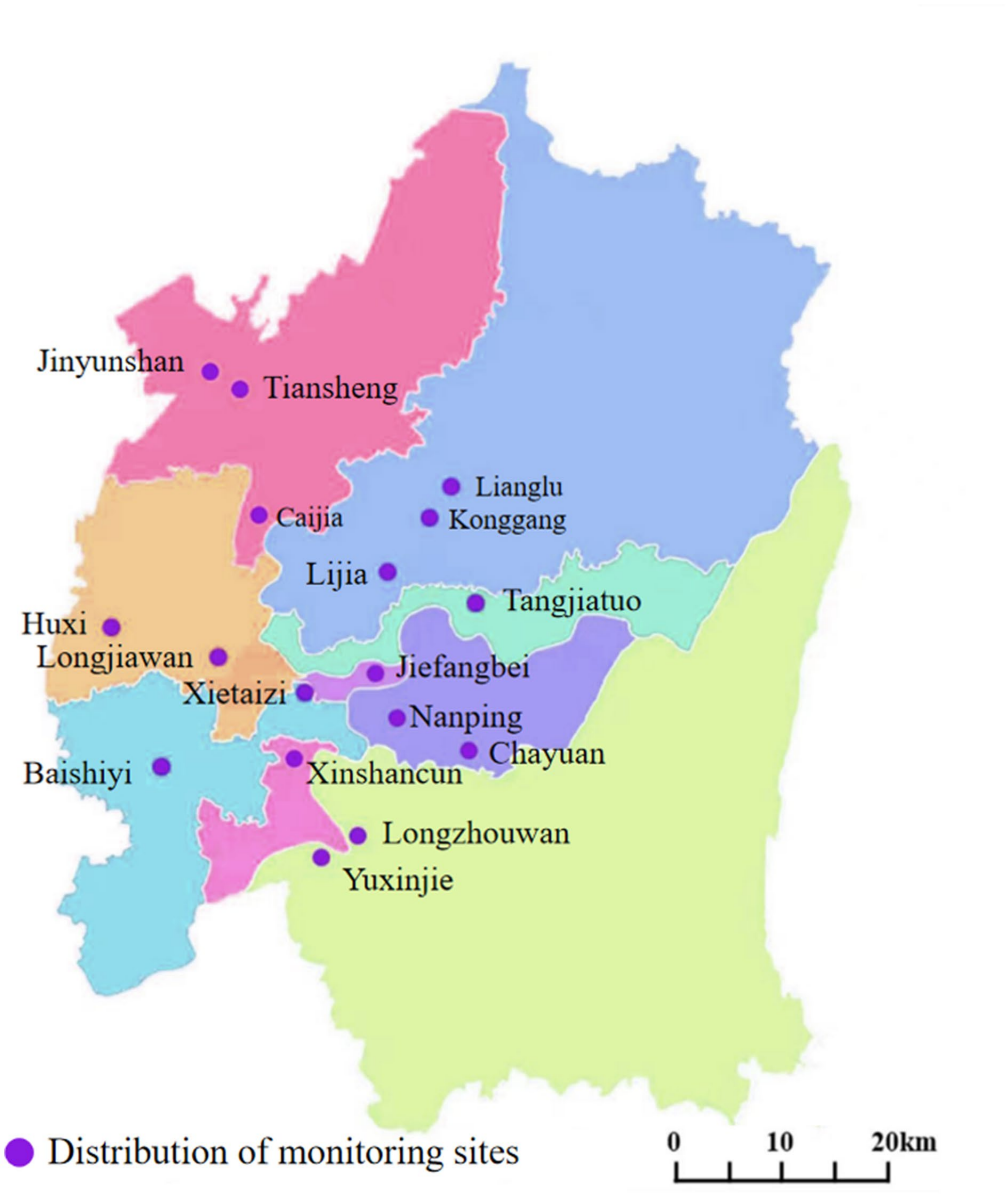
Locations of air quality-monitoring stations in the main urban areas of Chongqing.

The data for air pollutant levels in the main urban area of Chongqing were obtained from a report issued by the Environmental Protection Bureau. The time period was from December 22, 2018 to December 21, 2021. The report included data from the following 17 monitoring sites in the main urban area. The specific locations of the monitoring sites are shown in Figure 1. The average daily AQI and daily concentrations of PM_10_, PM_2.5_, SO_2_, NO_2_, CO and O_3_ were collected. AQI is a dimensionless index that is determined by taking the maximum values for air quality sub-indexes of PM_10_, PM_2.5_, SO_2_, NO_2_, O_3_, and CO from the Technical Regulation on Ambient Air Quality Index of China (NO: HJ633-2012) (19). The PM_10_, PM_2.5_, SO_2_, NO_2_, and O_3_ levels were reported in units of μg/m^3^, and the CO level as mg/m^3^. The average concentrations of PM_10_, PM_2.5_, SO_2_, NO_2_, and CO were obtained by averaging the hourly concentrations sampled 24 times a day. For O_3_, the 8-h maximum average in a day was used.

The meteorological data for Chongqing were collected between December 22, 2018 and December 21, 2021 from the China Meteorological Data Sharing Service System with a spatial resolution of 0.0625°× 0.0625°.^1^ The collected data included the daily average values for temperature, relative humidity, atmospheric pressure, and wind speed.

### 2.4. Study design and statistical analyses

The study employed a time-stratified, case-crossover design and can be viewed as a special case–control study. In this design, each case serves as its own control to control for the potential confounding influence of individual characteristics, such as age, sex, and family economic conditions. This method has been widely used in epidemiological studies to explore the risks of acute events (20). Considering that the medication period for AOM is 1–2 weeks after diagnosis, children will not seek treatment again even if air pollution levels increase during this time. As a result, we selected three control time points for each case of 2 weeks after and 1 and 2 weeks before the day of the emergency visit.

SPSS 23 software was used for statistical analyses of the case characteristics, air pollution variables, and meteorological variables. Correlation analysis was conducted between meteorological variables and air pollution variables. Pearson correlation test was used for data that followed a normal distribution, and Spearman test was used for data not consistent with a normal distribution. Then, a conditional logistic regression method was used with the daily number of patients as the weight through the Cox regression module in the SPSS software (21). Meteorological factors that could affect AOM visits and levels of air pollution variables were viewed as control variables in the model. The model was as follows:

$$\begin{array}{l} \ln\left(h\left(t,X\right)\right)=\ln\left(h_{0i}\left(t\right)\right)+T\beta_{1}+RH\beta_{2}+\\ AP\beta_{3}+WS\beta_{4}+C\left(AQI\right)\beta_{5}+\\ C\left({\mathrm{PM}}_{10}\right)\beta_{6}+C\left({\mathrm{PM}}_{2.5}\right)\beta_{7}+C\left({\mathrm{SO}}_{2}\right)\beta_{8}+\\ C\left(CO\right)\beta_{9}+C\left(NO2\right)\beta_{10}+C\left(O_{3}\right)\beta_{11} \end{array}$$

where, “t” refers to the day; “X” refers to the emergency visit; “ln (h (t, X))” refers to the risk function; “ln (h0i (t))” refers to the baseline risk function; “T, RH, AP and WS” refer to the temperature, relative humidity, atmospheric pressure and wind speed, respectively; “C (AQI), C (PM_10_), C (PM_2.5_), C (SO_2_),C (CO), C (NO_2_), C (O_3_)” are the corresponding values of AQI or the concentrations of air pollutants; and “*β*_1_*-β*_11_” refers to the coefficient for each covariate. Results were represented by the percentage change in the number of daily visits caused by an increase in AQI and air pollutant concentrations, in the form of odds ratios (ORs) and the corresponding 95% confidence intervals (CIs).

The health effects of air pollutants have a lag effect, and Wong et al. (22) reported that a single lag model may underestimate the health impact of air pollutants. Therefore, the present study used a lag model that included single-day lags from lag 0 to lag 7 and multi-day lags from lag 0–1 to lag 0–7. Lag 0 referred to the day on which AOM was diagnosed, and lag 1 corresponded to the previous day, up to lag 7. Lag 0–1 referred to the 2-day mean for the same day and previous day, and lag 0–7 referred to the 8-day mean for the same day and all days to 7 days before. The meteorological variables were also lagged when the pollution data lagged in a lagged model. In addition, the best lag periods for air pollution variables were determined according to the maximum values of odds ratios (ORs) in the single-pollutant model.

To adjust for the potential effect of modification such as gender, age and season on the results, cases in this study were stratified according to gender (male and female), age group (0, 1–2, 3–5, and 6–17 years), and season (Spring, Summer, Autumn, and Winter). The health effect values for air pollution variables were analyzed with stratification. The differences were tested for statistical significance by calculating 95% CIs based on the following formula:

$$\left({\hat{Q}}_{1}-{\hat{Q}}_{2}\right)\pm 1.96\sqrt{\;{\left(S{\hat{E}}_{1}\right)}^{2}+{\left(S{\hat{E}}_{2}\right)}^{2}}$$

where ${\hat{Q}}_{1}$ and ${\hat{Q}}_{2}$ are the effect estimates for the two categories, and $S{\hat{E}}_{1}$ and $S{\hat{E}}_{2}$ are their respective standard errors (23).

## 3. Results

### 3.1. Characteristics of pediatric patients with AOM

**Figure 2:**
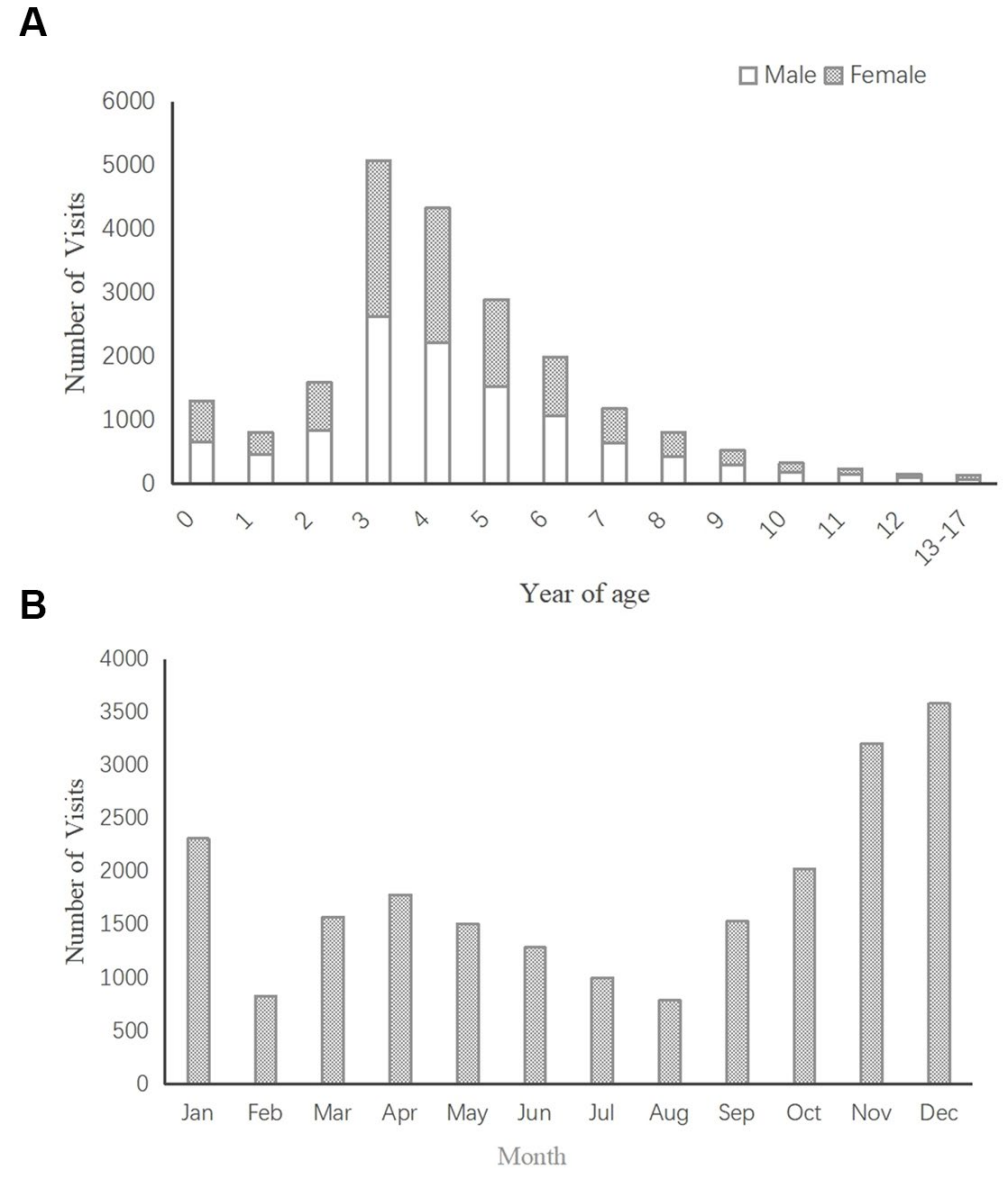
**(A)** Distribution of AOM visits by age (*n* = 21,416). **(B)** Distribution of AOM visits by month (*n* = 21,416).

A total of 21,416 children were included in this study, with a male to female ratio of 1.12:1 (11,329:10087). The median age at onset was 4.410 years (3.360 years, 6.015 years). Figure 2A shows the number of cases according to different ages. The 3- to 5-year age range included the largest number of patients, accounting for 57.49% of all patients (*n* = 12,311). Figure 2B shows the number of cases diagnosed in each month. The month in which the highest number of cases presented was December followed by November and then January.

### 3.2. Summary statistics for air pollution and meteorological variables

Table 1 shows the summarized data for air pollution variables (AQI, PM_10_, PM_2.5_, SO_2_, NO_2_, O_3_, and CO) and meteorological variables (mean temperature, relative humidity, atmospheric pressure, and wind speed) during the study period. The results show that the AQI value, the concentrations of PM_10_, PM_2.5_, CO, SO_2_, CO and NO_2_ were highest in Winter, whereas the highest concentrations of O_3_ occurred in Summer. Because these variables were not normally distributed, the Kruskal Wallis test was used to compare the values of variables among different seasons, and the results suggested that all observed group differences were statistically significant.

**Table 1:** Summary statistics for air pollution and meteorological variables (AQI is unitless; CO concentration is reported as mg/m^3^; and PM_10_, PM_2.5_, SO_2_, NO_2_ and O_3_ concentrations are reported as μg/m^3^).

Variables	All				Spring	Summer	Autumn	Winter
	Min	Max	Mean ± SD	IQR	Mean ± SD	Mean ± SD	Mean ± SD	Mean ± SD
AQI	21.00	203.00	66.69 ± 29.25	38.00	62.39 ± 23.47	68.79 ± 35.26	56.56 ± 21.05	79.12 ± 30.04
PM_10_	14.00	198.00	54.74 ± 38.52	35.75	54.58 ± 18.45	36.83 ± 13.27	48.07 ± 25.13	79.89 ± 33.45
PM_2.5_	8.00	143.00	34.96 ± 21.02	24.00	32.61 ± 11.15	20.78 ± 7.52	29.24 ± 15.93	57.56 ± 24.31
SO_2_	1.00	17.00	8.13 ± 2.28	3.00	8.11 ± 2.13	7.39 ± 1.85	8.13 ± 2.35	8.91 ± 2.47
CO	0.30	1.70	0.79 ± 0.178	0.20	0.75 ± 0.13	0.71 ± 0.12	0.76 ± 0.16	0.93 ± 0.20
NO_2_	9.00	76.00	36.32 ± 11.58	15.00	38.61 ± 10.67	30.22 ± 8.33	36.53 ± 12.17	39.97 ± 12.22
O_3_	5.00	277.00	72.27 ± 50.14	68.00	84.07 ± 45.25	111.95 ± 50.19	56.85 ± 41.00	35.36 ± 21.61
Temp	4.20	35.60	19.31 ± 7.58	13.58	19.29 + 4.57	28.18 ± 3.41	19.33 ± 5.55	10.28 ± 2.46
RH	39.80	97.00	75.80 ± 10.83	15.20	73.84 ± 10.37	72.52 ± 12.62	79.72 ± 10.11	77.19 ± 8.16
AP	965.00	1005.10	983.12 ± 8.87	15.40	981.85 ± 6.34	972.81 ± 3.21	986.38 ± 6.71	991.62 ± 5.34
WS	0.00	3.70	1.21 ± 0.42	0.50	1.29 ± 0.45	1.29 ± 0.45	1.12 ± 0.37	1.11 ± 0.36

Spring refers to March, April, and May; Summer refers to June, July, and August; Autumn refers to September, October, and November; and Winter refers to December, January, and February. Temp, temperature; RH, relative humidity; AP, atmospheric pressure; WS, wind speed.

### 3.3. Correlations between air pollution variables and meteorological variables

The Spearman test was used to identify correlations between different air pollution variables and meteorological variables. As shown by the data in Table 2, PM_10_ and PM_2.5_ were most strongly correlated (correlation coefficient = 0.965, *p* < 0.01).

**Table 2:** Spearman correlation coefficients for correlations between air pollution variables and meteorological variables.

	AQI	PM_10_	PM_2.5_	SO_2_	CO	NO_2_	O_3_	Temp	RH	AP	WS
AQI	1										
PM_10_	0.743**	1									
PM_2.5_	0.646**	0.965**	1								
SO_2_	0.543**	0.617**	0.531**	1							
CO	0.507**	0.653**	0.673**	0.264**	1						
NO_2_	0.576**	0.729**	0.657**	0.451**	0.691**	1					
O_3_	0.262**	−0.155**	−0.273**	0.060*	−0.264**	−0.153**	1				
Temp	0.005	−0.437**	−0.560**	−0.092**	−0.355**	−0.303**	0.760**	1			
RH	−0.514**	−0.274**	−0.134**	−0.470**	0.038	−0.163**	−0.629**	−0.344**	1		
AP	0.015	0.386**	0.474**	0.126**	0.255**	0.308**	−0.656**	−0.873**	0.267**	1	
WS	−0.099**	−0.145**	−0.157**	−0.225**	−0.116**	−0.126**	0.130**	0.119**	−0.126**	−0.158**	1

Temp, temperature; RH, relative humidity; AP, atmospheric pressure; WS, wind speed. *p < 0.05, **p < 0.01.

### 3.4. Single-pollutant models for the associations between air pollution variables and emergency visits for AOM in children

Table 3 shows the results from the single-pollutant models for air pollution variables after controlling for the meteorological factors. Increases in AQI and the concentrations of PM_10_, PM_2.5_, SO_2,_ CO and NO_2_ were significantly associated with an increased risk for emergency visits for AOM, while no significant association was detected for changes in the concentrations of O_3_. The strongest effect on emergency visits was observed on the lag 0–7 model except for O_3_, and the largest OR was 1.185 (95% CI, 1.129–1.245) for SO_2_.

**Table 3:** Associations between air pollution variables (per IQR increase in AQI and the concentrations of air pollutants) and emergency visits for AOM in children: single-pollutant models.

	AQI	PM_10_	PM_2.5_	SO_2_	CO	NO_2_	O_3_
L0	1.000 (0.975–1.025)	1.006 (0.984–1.028)	1.001 (0.980–1.022)	1.010 (0.983–1.038)	1.019 (0.997–1.041)	1.031* (1.004–1.057)	0.980 (0.935–1.028)
L1	1.018 (0.993–1.044)	1.019 (0.996–1.042)	1.015 (0.993–1.037)	1.017 (0.990–1.045)	1.021 (1.000–1.044)	1.049** (1.022–1.076)	0.968 (0.923–1.015)
L2	1.027* (1.002–1.053)	1.035** (1.012–1.058)	1.031** (1.009–1.053)	1.031* (1.003–1.059)	1.020 (0.999–1.042)	1.054** (1.028–1.082)	0.962 (0.918–1.009)
L3	1.063** (1.038–1.089)	1.059** (1.037–1.083)	1.057** (1.035–1.079)	1.072** (1.043–1.101)	1.047** (1.025–1.069)	1.080** (1.052–1.108)	1.017 (0.970–1.066)
L4	1.056** (1.031–1.082)	1.065** (1.042–1.089)	1.060** (1.039–1.083)	1.077** (1.049–1.107)	1.062** (1.040–1.085)	1.087** (1.060–1.116)	0.976 (0.931–1.023)
L5	1.071** (1.046–1.097)	1.074** (1.050–1.097)	1.072** (1.050–1.094)	1.088** (1.059–1.118)	1.065** (1.043–1.088)	1.097** (1.069–1.126)	0.971 (0.926–1.018)
L6	1.081** (1.055–1.107)	1.077** (1.053–1.101)	1.075** (1.053–1.098)	1.081** (1.052–1.111)	1.064** (1.041–1.086)	1.097** (1.069–1.126)	1.003 (0.957–1.050)
L7	1.092** (1.066–1.119)	1.084** (1.061–1.108)	1.081** (1.059–1.104)	1.085** (1.056–1.115)	1.065** (1.043–1.088)	1.111** (1.083–1.140)	0.985 (0.940–1.032)
L0-1	1.011 (0.983–1.038)	1.014 (0.989–1.038)	1.008 (0.986–1.032)	1.017 (0.985–1.050)	1.023 (0.999–1.048)	1.048** (1.019–1.078)	0.968 (0.915–1.024)
L0-2	1.020 (0.990–1.050)	1.024 (0.998–1.051)	1.018 (0.994–1.043)	1.029 (0.993–1.067)	1.025 (0.999–1.051)	1.060** (1.029–1.092)	0.954 (0.894–1.018)
L0-3	1.044** (1.012–1.077)	1.044** (1.016–1.072)	1.037** (1.011–1.064)	1.064** (1.024–1.107)	1.040** (1.012–1.068)	1.083** (1.049–1.117)	0.976 (0.908–1.050)
L0-4	1.059** (1.025–1.095)	1.059** (1.029–1.089)	1.037** (1.011–1.064)	1.095** (1.050–1.141)	1.040** (1.012–1.068)	1.102** (1.066–1.139)	0.973 (0.900–1.053)
L0-5	1.079** (1.043–1.116)	1.076** (1.044–1.108)	1.068** (1.039–1.098)	1.128** (1.080–1.179)	1.071** (1.040–1.103)	1.122** (1.085–1.161)	0.971 (0.892–1.056)
L0-6	1.097* (1.059–1.136)	1.089** (1056–1.123)	1.081** (1.050–1.112)	1.152** (1.100–1.206)	1.081** (1.049–1.115)	1.137** (1.097–1.177)	0.971 (0.892–1.056)
L0-7	1.123** (1.082–1.165)	1.107** (1.072–1.143)	1.098** (1.065–1.132)	1.185** (1.129–1.245)	1.097** (1.062–1.133)	1.159** (1.117–1.202)	0.971 (0.892–1.056)

L, lag. L0 refers to day of AOM diagnosis, and L1 corresponds to the previous day, up to L7. L0-1 refers to the 2-day mean for the same day and previous day, and L0-X refers to the X + 1-day mean for the same day and all days to X days before. *p < 0.05, **p < 0.01 adjusted for temperature, relative humidity, atmospheric pressure, and wind speed.

### 3.5. Multi-pollutant models for associations between air pollution variables and emergency visits for AOM in children

AQI is not one pollutant as PM_10_, PM_2.5_, SO_2_, NO_2_, O_3_, or CO. It is the maximum value reflecting the air quality sub-indexes for six pollutants. If we include AQI in multi-pollutant models, there might be overlapping effects. So we did not include the AQI in the multi-pollutant models. Table 2 shows the correlation coefficient for PM_10_ and PM_2.5_ was 0.965, and Table 3 shows PM_10_ had a higher OR value for influencing emergency visits for AOM. Therefore, we included PM_10_ and excluded PM_2.5_ in the multi-pollutant models, consistent with the approach used by Ding et al. and Ko et al. (24, 25). Table 4 shows that in the multi-pollutant models, most OR values for PM_10_, SO_2_, CO and NO_2_ were statistically significant. We found the statistically significant OR_S_ values for PM_10_ became smaller after adjusting for CO and became slightly bigger after adjusting for O_3_. For the statistically significant OR values for SO_2_, the ORs became smaller after adjusting for PM_10_ and CO, and the OR became slightly bigger after adjusting for O_3_. Also, the statistically significant OR values for CO became slightly bigger after adjusting for O_3_. For the statistically significant OR values for NO_2_, all the ORs became bigger except after adjusting for SO_2_. The OR values for NO_2_ were significant in all the multi-pollutant models and the OR values for O_3_ were not significant in any of the multi-pollutant models.

**Table 4:** Associations between air pollution variables (per IQR increase in AQI and the concentrations of air pollutants) and emergency visits for AOM in children: multi-pollutant models.

	PM_10_ (lag 0–7)	SO_2_ (lag 0–7)	CO (lag 0–7)	NO_2_ (lag 0–7)	O_3_ (lag 3)
Adjusted for PM_10_	/	1.131** (1.049–1.219)	1.035 (0.985–1.087)	1.168** (1.097–1.243)	1.014 (0.967–1.063)
Adjusted for SO_2_	1.041 (0.991–1.094)	/	1.038 (0.997–1.082)	1.125** (1.066–1.187)	1.018 (0.971–1.068)
Adjusted for CO	1.079* (1.028–1.133)	1.145** (1.076–1.218)	/	1.171** (1.108–1.237)	1.010 (0.963–1.059)
Adjusted for NO_2_	0.992 (0.939–1.047)	1.056 (0.982–1.135)	0.988 (0.941–1.038)	/	0.999 (0.953–1.048)
Adjusted for O_3_	1.109** (1.074–1.146)	1.188** (1.131–1.248)	1.099** (1.064–1.136)	1.167** (1.124–1.211)	/
Adjusted for the other four pollutants	0.973 (0.912–1.037)	1.069 (0.988–1.158)	0.991 (0.940–1.045)	1.162** (1.082–1.247)	1.005 (0.958–1.055)

*p < 0.05, **p < 0.01 adjusted for temperature, relative humidity, atmospheric pressure and wind speed.

### 3.6. Stratified analyses of the associations between air pollution variables and emergency visits for AOM in children

Table 5 shows the results of stratified analyses according to gender, age and season. The OR values for associations between O_3_ and AOM were not statistically significant with any gender and age stratification. With gender stratification, the OR values for the effects of increases in AQI, PM_10_, PM_2.5_, CO and NO_2_ on emergency visits for AOM were greater for male patients than for female patients, and the OR values for increases in SO_2_ were greater for female patients than for male patients. With age stratification, the statistically significant OR values for the effects of increases in AQI, PM_10_, SO_2_, and NO_2_ on emergency visits for AOM were greatest for patients aged 3–5 years, while the greatest OR values for PM_2.5_ and CO were observed for the groups aged 0 years, respectively. However, there was no difference between groups in these statistically significant OR values through gender and age stratification. With season stratification, the statistically significant OR values for AQI, PM_2.5_ and CO were highest in Winter, the statistically significant OR values for PM_10_, SO_2_ and NO_2_ were highest in Spring; and the statistically significant OR values for O_3_ were highest in Summer. Notably, the differences between seasons in these statistically significant OR values of PM_10_, SO_2_, CO, NO_2_ and O_3_ were statistically significant.

**Table 5:** Associations between air pollution variables (per IQR increase in AQI and the concentrations of air pollutants) and emergency visits for AOM in children upon stratification by patient characteristics.

	AQI	PM_10_	PM_2.5_	SO_2_	CO	NO_2_	O_3_
Sex							
Male	1.135** (1.078–1.195)	1.115** (1.067–1.165)	1.100** (1.055–1.147)	1.181** (1.104–1.263)	1.109** (1.061–1.160)	1.186** (1.127–1.247)	1.019 (0.955–1.086)
Female	1.109** (1.051–1.171)	1.098** (1.048–1.151)	1.096** (1.049–1.145)	1.190** (1.108–1.278)	1.082** (1.033–1.135)	1.130** (1.071–1.192)	1.015 (0.947–1.089)
Age (years)							
0	1.133 (0.975–1.316)	1.144 (1.000–1.309)	1.159* (1.021–1.317)	1.206 (0.974–1.492)	1.155* (1.007–1.325)	1.112 (0.962–1.286)	1.058 (0.891–1.257)
1–2	1.052 (0.944–1.173)	1.083 (0.985–1.190)	1.061 (0.971–1.159)	1.206* (1.039–1.401)	1.104* (1.004–1.215)	1.127* (1.014–1.253)	0.985 (0.857–1.132)
3–5	1.133** (1.079–1.190)	1.112** (1.066–1.159)	1.102** (1.059–1.146)	1.207** (1.133–1.286)	1.094** (1.049–1.141)	1.186** (1.130–1.245)	1.063 (0.996–1.135)
6–17	1.129** (1.049–1.215)	1.108** (1.037–1.184)	1.099** (1.033–1.169)	1.134* (1.025–1.253)	1.090* (1.020–1.166)	1.140** (1.057–1.229)	0.936 (0.856–1.024)
Season							
Spring	0.889 (0.763–1.036)	1.400** (1.215–1.613)	1.053 (0.899–1.235)	1.500** (1.261–1.785)	0.825** (0.715–0.953)	1.556** (1.387–1.746)	1.099 (0.994–1.215)
Summer	1.139 (0.968–1.341)	1.037 (0.782–1.376)	0.902 (0.666–1.222)	1.413** (1.116–1.790)	0.950 (0.789–1.144)	1.272* (1.025–1.577)	1.167** (1.060–1.285)
Autumn	0.908 (0.817–1.010)	1.127** (1.046–1.214)	1.008 (0.937–1.085)	1.039 (0.938–1.152)	1.045 (0.973–1.123)	1.392** (1.293–1.498)	1.010 (0.926–1.102)
Winter	1.175** (1.120–1.233)	1.115** (1.071–1.161)	1.154** (1.112–1.197)	1.306** (1.224–1.393)	1.283** (1.230–1.338)	1.147** (1.090–1.208)	0.795** (0.693–0.911)

*p < 0.05, **p < 0.01 adjusted for temperature, relative humidity, atmospheric pressure and wind speed.

## 4. Discussion

In one of the first studies on this topic in Asia, the present study analyzed the short-term effects of air pollution on pediatric emergency visits for AOM by collecting data for seven air pollutant variables from 17 monitoring sites in Chongqing, China. Data for a total of 21,416 children aged 0–17 years were collected for this study, representing the largest sample size among the relevant published studies from a single center. A 1:3 case-crossover design was used to control individual characteristics. Additionally, data for meteorological variables were collected as confounding factors.

The results of this study found that in the single-pollutant and stratified analyses increases in the AQI as well as the concentrations of PM_10_, PM_2.5_, SO_2_, CO and NO_2_ led to statistically significant increases in the number of pediatric AOM cases, and in multi-pollutant models, increases in the concentrations of PM_10_, SO_2_, CO and NO_2_ led to statistically significant increases in the number of pediatric AOM cases. These findings are consistent with most epidemiological studies (12, 26–31). However, a few studies did not find short-term associations between these air pollution variables and AOM. Although a case-crossover study from Windsor, Ontario, Canada reported a significant association between PM_2.5_ and emergency department visits for AOM in children, they found no significant association between SO_2_, CO and NO_2_ levels and AOM presentations (32). Strickland et al. (33) did not find significant associations between emergency department visits for AOM and same-day and previous-day PM_2.5_ concentrations through time-stratified case-crossover models stratified by ZIP code, year, and month. Moreover, another Canadian study analyzing data collected over 10 years also used a case-crossover design to explore the association between emergency department visits for OM and air pollution through a sample of 14,527 children aged 1–3 years (13). In their study, the ORs for PM_10_, CO, NO_2_ and O_3_ were positive statistically significant, while those for SO_2_ and PM_2.5_ were not positive statistically significant.

In the present study, no significant associations were found between increases in O_3_ concentrations and the number of patients with AOM in the single-pollutant model and multi-pollutant models. Similarly, a time series study from Lanzhou, China analyzed the correlation between environment-meteorological factors and patients with AOM in entire population (34). The results showed that PM_2.5_, PM_10_, NO_2_, SO_2_, and CO are positively correlated with daily visits to AOM, whereas O_3_ is not. Surprisingly, we found that the OR values for O_3_ were statistically significant upon seasonal stratification. However, several other studies did report significant ORs for the effects of O_3_ concentrations on AOM (12, 13, 27, 32). Therefore, the associations of O_3_ with AOM need to be confirmed by more epidemiological studies, and seasonal stratification should be carried out if necessary.

In our single pollutant models, we found that the best lag periods were lag 0–7 for air pollution variables except O_3_. This best period was later than other best periods in some studies (13, 28, 32, 34). One possible reason is that most of these studies did not use cumulative lags, which are more robust and significant than individual lags (13, 28, 32). Another possible reason is that otitis media is often secondary to upper respiratory infection (URI) (31), and thus, the best lag period may be late. However, since the effects would be overlapping and mixed, we did not continue to analyze the effect values after lag 7 and lag 0–7. More studies are needed to explore the best lag periods for air pollution effecting emergency visits for AOM.

Although the results of multi-pollutant models showed that PM_10_, SO_2_, CO and NO_2_ were positively correlated with emergency visits to AOM after controlling some air pollution variables, only the OR values for NO_2_ were statistically significant in each multi-pollutant model. Also, NO_2_ had the most statistically significant OR values in the single-pollutant models and stratified analyses. These results indicate that NO_2_ is the most significant pollutant variable in our study. NO_2_ was associated with the visits for OM in other studies, but it has not had the most significant OR values (13, 27). In the largest birth cohort studies involving 10 European birth cohorts, a significant positive association was found between NO_2_ and OM, while no significant association was found between PM_10_, PM_2.5_ and OM (9). These results indicate that NO_2_ has more robust effects on OM than PM_10_ and PM_2.5_. The reason maybe NO_2_ can impair the mucociliary clearance of the upper respiratory tract and middle ear and alter the inflammatory response to infections, possibly resulting in an increased number of visits for URI and OM (30, 35, 36).

The distribution of patients according to age (Figure 2A) showed that the peak incidence of AOM occurred in preschool children aged 3–5 years, which is not completely consistent with other studies (5, 17). With age stratification, our results showed that the highest OR values were aged 0 years or 3–5 years or for air pollution variables. These results indicate that children aged 0 years and 3–5 years may be the most susceptible group to air pollution. One possible explanation is that the immunity of children less than 5 years of age is weaker than that of older children. Another possible reason is that these children may spend more time outdoors, leading to more chances for inhalation of air pollutants. Lastly, children less than 5 years old have the peak incidence of URI, and air pollution can increase the incidence of URI (37, 38). Approximately 35% of pediatric cases of URI are complicated by OM (39, 40). Finally, air pollution indirectly increases the incidence of AOM in children less than 5 years old.

In the present study, November, December and January had the highest AOM incidence (Figure 2B), which is similar to the results of most previous epidemiological studies (27, 41). November in within Autumn, and December and January are in Winter in Chongqing. Our seasonal stratification results showed that the positive significant OR values for AQI, PM_10_, PM_2.5_, SO_2_, CO and NO_2_ were highest in Winter or Spring. The results indicate that children are most affected by air pollution during Winter and Spring. One explanation may be that heavy pollution combined with the low temperatures lead to the highest incidence of respiratory infection, which is an important cause of AOM in children.

As an environmental epidemiological study, the present study still has some limitations. First, the pollution concentrations we studied came from areas of children’s residences. Although children spend much time in school, China has the policy of going to school near to one’s residence, and thus, children will go to the school that the closest to their home, making the error between the pollution concentrations at their school and residence extremely small. However, the children’s actual exposure levels to air pollution likely differ from the measured pollution concentrations due to differences in indoor environments, personal outdoor activity habits, and so on. Therefore, there is deviation between the exposure level, and it is difficult to estimate the magnitude and direction of this deviation. Second, due to technical limitations and the small area of Chongqing’s main urban area, which is only 4,779 km^2^, the data for air pollution variables were averaged from 17 fixed monitoring stations. The results would be more accurate if concentrations of air pollutants could be obtained according the children’s specific residential locations. For example, Xiao et al. (12) used CMAQ model simulations and ground-based measurements to estimate the concentrations of air pollutants, and Ko et al. (25) modeled the concentration PM_2.5_ through satellite, remote sensing, meteorological and land use data. In addition, compared with other multi-center or national studies in developed countries, this study was only a single-center study. Our findings may not be applicable to other cities and populations.

In conclusion, increases in the AQI and the concentrations of PM_10_, PM_2.5_, SO_2,_ CO and NO_2_ were positively associated with emergency visits for AOM by children. However, increases in O_3_ showed effects on AOM visits just in summer. We found that the best lag periods were lag 0–7 for air pollution variables except O_3_ in single pollutant models. As NO_2_ had the most statistically significant OR values in all models of our study, we need to control car exhaust to reduce NO_2_. Children aged 0 years and 3–5 years were most susceptible to the effects of air pollution on the occurrence of AOM, and Winter and Spring were the seasons when air pollutant levels had the most positive significant effects on AOM visits. These findings can provide a basis for the early prevention in Winter and Spring and for susceptible children to prevent the occurrence of AOM. Further multi-center studies are needed, particularly using more accurate measurement of exposure levels, to explore the effects of air pollution on AOM.

## Data availability statement

The original contributions presented in the study are included in the article/supplementary material, further inquiries can be directed to the corresponding author.

## Ethics statement

The research protocol was approved by the Medical Ethics Committee of the Children’s Hospital of the Chongqing Medical University. Informed consent was obtained from all individual participants and/or their legal guardians included in the study.

## Author contributions

LX collected data and wrote the paper. SS finished statistical analysis. CC and HY continued to check data and paper, and LD proposed ideas and finished project administration. All authors contributed to the article and approved the submitted version.

## Footnotes

^1^ http://data.cma.cn/

## References

[1] Ahmed S Shapiro NL Bhattacharyya N. Incremental health care utilization and costs for acute otitis media in children. Laryngoscope. (2014) 124:301–5. doi: 10.1002/lary.24190, PMID: 23649905

[2] Bowatte G Tham R Perret JL Dharmage SC Dong G Waidyatillake N. Air pollution and otitis media in children: a systematic review of literature. Int J Environ Res Public Health. (2018) 15:257. doi: 10.3390/ijerph15020257, PMID: 29401661 PMC5858326

[3] Leung AKC Wong AHC. Acute otitis media in children. Recent Pat Inflamm Allergy Drug Discov. (2017) 11:32–40. doi: 10.2174/1874609810666170712145332 28707578

[4] Bateson TF Schwartz J. Children’s response to air pollutants. J Toxicol Environ Health. (2008) 71:238–43. doi: 10.1080/15287390701598234 18097949

[5] Ohashi Y Nakai Y Furuya H Esaki Y Ikeoka H Kato S. Mucociliary disease of the middle ear during experimental otitis media with effusion induced by bacterial endotoxin. Ann Otol Rhinol Laryngol. (1989) 98:479–84. doi: 10.1177/000348948909800616, PMID: 2658718

[6] Song JJ Lee JD Lee BD Chae SW Park MK. Effect of acrolein, a hazardous air pollutant in smoke, on human middle ear epithelial cells. Int J Pediatr Otorhinolaryngol. (2013) 77:1659–64. doi: 10.1016/j.ijporl.2013.07.021, PMID: 23953484

[7] Coticchia JM Chen M Sachdeva L Mutchnick S. New paradigms in the pathogenesis of otitis media in children. Front Pediatr. (2013) 1:52. doi: 10.3389/fped.2013.00052, PMID: 24400296 PMC3874850

[8] Shusterman D. The effects of air pollutants and irritants on the upper airway. Proc Am Thorac Soc. (2011) 8:101–5. doi: 10.1513/pats.201003-027RN 21364227

[9] MacIntyre EA Gehring U Molter A Fuertes E Klumper C Kramer U. Air pollution and respiratory infections during early childhood: an analysis of 10 European birth cohorts within the ESCAPE Project. Environ Health Perspect. (2014) 122:107–13. doi: 10.1289/ehp.1306755, PMID: 24149084 PMC3888562

[10] Brauer M Gehring U Brunekreef B de Jongste J Gerritsen J Rovers M. Traffic-related air pollution and otitis media. Environ Health Perspect. (2006) 114:1414–8. doi: 10.1289/ehp.9089, PMID: 16966098 PMC1570088

[11] Kennedy CM Pennington AF Darrow LA Klein M Zhai X Bates JT. Associations of mobile source air pollution during the first year of life with childhood pneumonia, bronchiolitis, and otitis media. Environ Epidemiol. (2018) 2:e007. doi: 10.1097/EE9.0000000000000007, PMID: 30215038 PMC6133251

[12] Xiao Q Liu Y Mulholland JA Russell AG Darrow LA Tolbert PE. Pediatric emergency department visits and ambient air pollution in the U.S. State of Georgia: a case-crossover study. Environ Health. (2016) 15:115. doi: 10.1186/s12940-016-0196-y, PMID: 27887621 PMC5124302

[13] Zemek R Szyszkowicz M Rowe BH. Air pollution and emergency department visits for otitis media: a case-crossover study in Edmonton, Canada. Environ Health Perspect. (2010) 118:1631–6. doi: 10.1289/ehp.0901675, PMID: 20663739 PMC2974705

[14] Deng Q Lu C Jiang W Zhao J Deng L Xiang Y. Association of outdoor air pollution and indoor renovation with early childhood ear infection in China. Chemosphere. (2017) 169:288–96. doi: 10.1016/j.chemosphere.2016.11.079, PMID: 27883914

[15] Deng Q Lu C Li Y Chen L He Y Sundell J. Association between prenatal exposure to industrial air pollution and onset of early childhood ear infection in China. Atmos Environ. (2017) 157:18–26. doi: 10.1016/j.atmosenv.2017.03.005

[16] Zhang C Wu W Gu J Han H Wang G Zhou L. Correlations between air pollution and number of patients of acute otitis media and acute tonsillitis in children. J Prev Med Chin PLA. (2017) 35:218–21. doi: 10.13704/j.cnki.jyyx.2017.03.006

[17] Wang X Wang M Xu B Wang J Zhong C. Correlation between acute otitis media in children and environmental meteorological factors. Chin J Otol. (2020) 18:527–33. doi: 10.3969/j.issn.1672-2922.2020

[18] Zhang L Huang C Wang Y Tan L. Correlation between air pollution and incidence of acute otitis and acute tonsillitis in children. J Kunming Med Univ. (2019) 40:78–81.

[19] Pu H Luo K Wang P Wang S Kang S. Spatial variation of air quality index and urban driving factors linkages: evidence from Chinese cities. Environ Sci Pollut R. (2017) 24:4457–68. doi: 10.1007/s11356-016-8181-0, PMID: 27943141

[20] Janes H Sheppard L Lumley T. Case-crossover analyses of air pollution exposure data: referent selection strategies and their implications for bias. Epidemiology. (2015) 16:717–26. doi: 10.1097/01.ede.0000181315.18836.9d, PMID: 16222160

[21] Pan B Zhang W Zhang X Wu S. Study on fitting conditional logistic regression model by using SPSS package. Strait J Prev Med. (2002) 8:1–4. doi: 10.3969/j.issn.1007-2705.2002.06.001

[22] Wong CM Vichit-Vadakan N Kan H Qian Z. Public health and air pollution in Asia (PAPA): a multicity study of short-term effects of air pollution on mortality. Environ Health Perspect. (2008) 116:1195–202. doi: 10.1289/ehp.11257, PMID: 18795163 PMC2535622

[23] Payton M Greenstone M Schenker N. Overlapping confidence intervals or standard error intervals: what do they mean in terms of statistical significance? J Insect Sci. (2003) 3:34. doi: 10.1093/jis/3.1.34 15841249 PMC524673

[24] Ding L Zhu D Peng D Zhao Y. Air pollution and asthma attacks in children: a case-crossover analysis in the city of Chongqing, China. Environ Pollut. (2017) 220:348–53. doi: 10.1016/j.envpol.2016.09.070, PMID: 27692885

[25] Ko FW Tam W Wong TW Lai CK Wong GW Leung TF. Effects of air pollution on asthma hospitalization rates in different age groups in Hong Kong. Clin Exp Allergy. (2007) 37:1312–9. doi: 10.1111/j.1365-2222.2007.02791.x, PMID: 17845411

[26] Park M Han J Park J Jang MJ Park MK. Particular matter influences the incidence of acute otitis media in children. Sci Rep. (2021) 11:19730. doi: 10.1038/s41598-021-99247-3, PMID: 34611241 PMC8492675

[27] Park M Han J Jang MJ Suh MW Lee JH Oh SH. Air pollution influences the incidence of otitis media in children: a national population based study. PLoS One. (2018) 13:e0199296. doi: 10.1371/journal.pone.0199296, PMID: 29953484 PMC6023207

[28] Girguis MS Strickland MJ Hu X Liu Y Chang HH Kloog I. Exposure to acute air pollution and risk of bronchiolitis and otitis media for preterm and term infants. J Expo Sci Env Epid. (2018) 28:348–57. doi: 10.1038/s41370-017-0006-9, PMID: 29269754 PMC6013343

[29] Oh J Lee S Kim MH Kwag Y Kim HS Kim S. The impact of PM_2.5_ on acute otitis media in children (aged 0–3): a time series study. Environ Int. (2020) 145:106133. doi: 10.1016/j.envint.2020.106133, PMID: 33011548

[30] Gestro M Condemi V Bardi L Fantino C Solimene U. Meteorological factors, air pollutants, and emergency department visits for otitis media: a time series study. Int J Biometeorol. (2017) 61:1749–64. doi: 10.1007/s00484-017-1356-7, PMID: 28466414

[31] Lee SY Jang MJ Oh SH Lee JH Suh MW Park MK. Associations between particulate matter and otitis media in children: a meta-analysis. Int J Environ Res Public Health. (2020) 17:4604. doi: 10.3390/ijerph17124604, PMID: 32604870 PMC7345266

[32] Kousha T Castner J. The air quality health index and emergency department visits for otitis media. J Nurs Scholarsh. (2016) 48:163–71. doi: 10.1111/jnu.12195 26836221

[33] Strickland MJ Hao H Hu X Chang HH Liu Y. Pediatric emergency visits and short-term changes in PM_2.5_ concentrations in the U.S. state of Georgia. Environ Health Persp. (2016) 124:690–6. doi: 10.1289/ehp.1509856, PMID: 26452298 PMC4858390

[34] Tian H Xu B Wang X Wang J Zhong C. Study on the correlation between ambient environment-meteorological factors and the number of visits of acute otitis media, Lanzhou, China. J Otol. (2020) 15:86–94. doi: 10.1016/j.joto.2020.01.002, PMID: 32884558 PMC7451727

[35] Frampton MW Boscia J Roberts NJ Azadniv M Torres A Cox C. Nitrogen dioxide exposure: effects on airway and blood cells. Am J Physiol Lung Cell Mol Physiol. (2002) 282:L155–65. doi: 10.1152/ajplung.2002.282.1.L155, PMID: 11741827

[36] Chauhan AJ Johnston SL. Air pollution and infection in respiratory illness. Brit Med Bull. (2003) 68:95–112. doi: 10.1093/bmb/ldg022 14757711

[37] Cheng J Su H Xu Z. Intraday effects of outdoor air pollution on acute upper and lower respiratory infections in Australian children. Environ Pollut. (2021) 268:115698. doi: 10.1016/j.envpol.2020.115698, PMID: 33049483

[38] Darrow LA Klein M Flanders WD Mulholland JA Tolbert PE Strickland MJ. Air pollution and acute respiratory infections among children 0-4 years of age: an 18-year time-series study. Am J Epidemiol. (2014) 180:968–77. doi: 10.1093/aje/kwu234, PMID: 25324558 PMC4224364

[39] Chonmaitree T Revai K Grady JJ Clos A Patel JA Nair S. Viral upper respiratory tract infection and otitis media complication in young children. Clin Infect Dis. (2008) 46:815–23. doi: 10.1086/528685, PMID: 18279042 PMC2744371

[40] Kalu SU Ataya RS McCormick DP Patel JA Revai K Chonmaitree T. Clinical spectrum of acute otitis media complicating upper respiratory tract viral infection. Pediatr Infect Dis J. (2011) 30:95–9. doi: 10.1097/INF.0b013e3181f253d5, PMID: 20711085 PMC3010420

[41] Heinrich J Raghuyamshi VS. Air pollution and otitis media: a review of evidence from epidemiologic studies. Curr Allergy Asthma Rep. (2004) 4:302–9. doi: 10.1007/s11882-004-0075-4, PMID: 15175145

